# Non-Pharmacological Therapeutic Options for the Treatment of Alzheimer’s Disease

**DOI:** 10.3390/ijms231911037

**Published:** 2022-09-20

**Authors:** Quy-Susan Huynh, Shalini Elangovan, R. M. Damian Holsinger

**Affiliations:** 1 Laboratory of Molecular Neuroscience and Dementia, School of Medical Sciences, Faculty of Medicine and Health, The University of Sydney, Camperdown, NSW 2050, Australia; 2 Neuroscience, School of Medical Sciences, Faculty of Medicine and Health, The University of Sydney, Sydney, NSW 2006, Australia

**Keywords:** photobiomodulation, fecal microbiota transplantation, deep brain stimulation

## Abstract

Alzheimer’s disease is a growing global crisis in need of urgent diagnostic and therapeutic strategies. The current treatment strategy mostly involves immunotherapeutic medications that have had little success in halting disease progress. Hypotheses for pathogenesis and development of AD have been expanded to implicate both organ systems as well as cellular reactions. Non-pharmacologic interventions ranging from minimally to deeply invasive have attempted to address these diverse contributors to AD. In this review, we aim to delineate mechanisms underlying such interventions while attempting to provide explanatory links between the observed differences in disease states and postulated metabolic or structural mechanisms of change. The techniques discussed are not an exhaustive list of non-pharmacological interventions against AD but provide a foundation to facilitate a deeper understanding of the area of study.

## 1. Introduction

Alzheimer’s disease (AD) is the most prevalent cause of dementia globally, with patients suffering from progressive impairment of cognition that ultimately impedes daily activities [1]. The primary pathological hallmarks of AD are the aggregation of amyloid beta (Aβ) peptides and accumulation of hyperphosphorylated neuronal tau proteins to form neurofibrillary tangles and subsequent chronic inflammation of the brain [2]. Genetic factors of AD vary depending on the subtype of the disease, such as the presence of the ε4 variant of the apolipoprotein E (APOE) gene that is associated with the more common sporadic form of AD (sAD) that manifests in those over the age of 65, or mutations in the amyloid precursor protein (APP) and presenilin (PSEN) genes that bring about the early onset of Alzheimer’s [3,4,5,6]. A range of causal mechanisms have been suggested to contribute to AD, including neuroinflammation [7], infectious agents [8], synaptic metabolic failure [9], and diabetes and obesity [10]. Urgent attention is required to manage, attenuate or possibly cure AD as it is poised to be the next health crisis. Numerous possible therapeutic agents have been proposed as a means to curb the extensive effects of AD. In this review, we evaluate non-pharmacological therapeutic interventions and list them, ranging from the least to the most invasive. In presenting such a format, we hope to provide the reader with alternative therapeutic strategies have been investigated in the realm of Alzheimer’s research. This review does not present an exhaustive listing of non-pharmacological interventions but rather is aimed at pointing the reader to the vast body of literature that is available in the field.

## 2. Noninvasive

### 2.1. Photobiomodulation

Photobiomodulation (PBM), formerly known as low-level laser (light) therapy (LLLT), utilizes laser or light as a noninvasive form of treatment. Although red or near-infrared (NIR) light has been the focus of many investigations, wavelengths of light from the visible (380–720 nm) [11] to the infrared spectrum (600–100 nm) [12] have been employed as a form of phototherapy where light is absorbed by chromophores present within cells. The absorbed light activates a chain of reactions that eventually leads to the upregulation of transcription factors involved in numerous cellular processes tasked with protecting the cell. While the application of PBM to the body is a relatively easy task, application to the nervous system, especially the brain, is more challenging. Some success has been achieved when applied through a sinus [13] or via a transcranial approach [14]. PBM wavelengths can penetrate up to 5 cm depending on the selected wavelength [15,16,17,18].

Although Aβ and neurofibrillary tangles are hallmark AD pathologies, patients also exhibit mitochondrial dysfunction, deficits in energy metabolism, increased reactive oxygen species (ROS), and neuroinflammation. Preclinical as well as clinical studies have demonstrated that PBM has the capability not only to address these issues, but also exert neuroprotective effects and therefore has a potential role in preventing AD progression.

#### 2.1.1. Mechanisms of Action

As the mitochondrial chromophore cytochrome c oxidase (CCO), otherwise known as complex IV, is one of a few proteins that absorbs light in the near-infrared spectrum and is also associated with energy metabolism, the enzyme has been shown to play a significant role in PBM. CCO exerts its action in the final step of the electron transport chain where it mediates the transfer of an electron from cytochrome c to molecular oxygen [19]. This process results in the increase in mitochondrial membrane potential and adenosine triphosphate (ATP) while modulating levels of reactive oxygen species (ROS) and inducing various transcription factors involved in key protein synthesis. A study evaluating the benefits of applying PBM at various wavelengths to rat postnatal visual cortex neurons, which previously were exposed to potassium cyanide, found that wavelengths of 670 nm and 880 nm were the most therapeutic [20]. These wavelengths coincide with the absorption range of oxidized CCO. Similarly, Wang and colleagues discovered that PBM treatment at 660 nm and 810 nm increased ATP levels and mitochondrial membrane potential whereas wavelengths of 415 nm (blue) and 540 nm (green) had an opposite effect [21]. Strong evidence demonstrating that PBM is capable of inducing changes in mitochondrial membrane potential, levels of ROS as well as ATP, supports the premise that CCO is involved in the underlying mechanisms of PBM [22,23].

Although the involvement of CCO is a prominent theory of investigations examining the effects of PBM, there is evidence suggesting that CCO may not be the key element. A study employing two genetically modified cell lines deficient in their ability to produce CCO found that both cell lines were still able to display benefits such as increased ATP synthesis and cell proliferation following PBM treatment at 660 nm [24]. An emerging theory also asserts the importance of water. Kim suggests the involvement of the fourth phase of water, referred to as the exclusion zone, and the nature of its interaction with hydrophilic surfaces such as biological molecules [25]. Similarly, Sommer and colleagues theorize that mitochondrial-bound water, in particular, is specifically significant to the mechanisms underlying PBM [26]. Other theories involve the action of light-gated ion channels [27] or propose that PBM has a systemic effect, given that studies have discovered therapeutic responses in regions of the body remote to the site of treatment. As an example, Fafara and colleagues irradiated mesenchymal stem cells in the tibia of mice and discovered a 68% reduction in Aβ deposition in the hippocampus [28].

#### 2.1.2. Effects of PBM on Amyloid Beta and Tau

The body of literature on the effects of PBM in AD is limited and mainly consists of investigations in either cellular or mouse models of the disease. Considering the potential of tau to influence normal tissue, Comerota and colleagues treated 12-month-old transgenic mice with NIR light (670 nm, 4 J/cm^2^ of light energy) for 90 s daily, 5 days/week for 4 weeks and discovered reduced tau and Aβ pathology in cortical and hippocampal synaptosomal extracts that translated to improved long-term memory in the treated animals [12]. These improvements were partially facilitated by activation of the heat shock and autophagy pathways [12], demonstrating once again that activation of clearance mechanisms may hold the key to treatment for the disease. A second study employing the more disease-aggressive 5xFAD mouse model of Alzheimer’s where plaque seeding occurs as early as two months of age also reported beneficial effects of PBM. Cho and colleagues treated 2- and 6-month-old 5xFAD mice for 20 min a day, 3 times per week for a period of 14 weeks (610 nm, 1.7 mW/cm^2^; 2.0 J/cm^2^). Following treatment, all mice were tested at 10 months of age in the Morris water maze (long-term and spatial memory), elevated plus maze (anxiety), and passive avoidance task (short- and long-term memory) [29]. Results demonstrated that PBM delivered at the very early stages of amyloid deposition exerts positive effects on both memory and pathology ~4.5 months following treatment. Mice treated at 2 months demonstrated near normal spatial-, short-, and long-term memory when compared to wild-type littermates and also displayed significantly reduced amyloid plaque load and microgliosis in their brains [29]. The results also demonstrated that treating mice at 2 months of age compared to 6 months yielded better results [29] but this could probably be attributed to the accumulation of amyloid from 2 to 6 months and the burden of toxicity and inflammation endured during that time. Furthermore, although levels of APP, BACE1, and neprilysin (an Aβ degrading enzyme) were unchanged, there was a significant increase of insulin-degrading enzyme (IDE), another enzyme capable of degrading Aβ. This set of results suggests that even though PBM may not prevent the formation of Aβ, treatment promotes Aβ degradation through IDE [29].

Changes in power densities and type of PBM doses have also been shown to differentially affect amyloid load [14]. Employing a wavelength of 808 ± 10 nm, De Taboada and colleagues demonstrated that pulsed treatments (100 Hz, 2 ms duration) outperformed continuous treatments in efficacy with pulsed treatments at a power density of 2830 mW/cm^2^ at the surface of the skin (50 mW/cm^2^ at the cortical surface), eliciting better outcomes compared to power densities of 566 mW/cm^2^ and 5660 mW/cm^2^ at the skin surface (10 mW/cm^2^ and 100 mW/cm^2^, respectively, at the cortical surface). The volume of brain amyloid was decreased by 67% at a density of 2830 mW/cm^2^, 54% at 566 mW/cm^2^, and 37.3% at 5660 mW/cm^2^. Brain levels of Aβ_1–40_ and the more fibrillogenic Aβ_1–42_ were also significantly decreased by pulsed PBM treatments [14].

PBM has also been shown to be efficacious in other mouse models of disease, including the K3 tau transgenic and the APPswe/PSEN1dE9 (APP/PS1) models [30]. Five-month-old APP/PS1 and 7-month-old K3 mice were exposed to a 670 nm NIR light source (44 mW/cm^2^ delivered to the skull) for a period of 90 s, 5 days/week for 4 weeks. Immunohistochemical analysis revealed significant decreases in hyperphosphorylated tau in the neocortex and hippocampus of K3 mice [30]. In addition to changes in tau, Purushothuman and colleagues also observed significant decreases in the oxidative stress markers 4-HNE and 8-OHDG [30]. PBM was also effective in regulating Aβ levels in the APP/PS1 mouse brain where plaques, gliosis, and synaptic loss was evident at around 4 months of age and cognitive decline emerged between 6 and 10 months and worsened with age. Here the researchers discovered a significant reduction in the percentage plaque burden, average plaque size, and the number of plaques in both the neocortex and the hippocampus [30].

Employing the TASTPM mouse, a different APP/PS1 model of AD where plaque pathology, gliosis, and cognitive impairment are observed at around 6 months of age, Grillo and colleagues delivered a 1072 nm wavelength light (pulsed at 600 Hz with a duty cycle of 300 μs, 5 mW/cm^2^) in 6-minute sessions for two consecutive days, bi-weekly for 5 months [31]. Evaluation of mice brains (n = 3) at 7 months of age revealed significant increases in the levels of a number of heat shock proteins (Hsps) and a reduction in Aβ levels in the brain. Hsps are a large family of molecular chaperones that play roles in protein maturation, refolding, and degradation. They form complexes with family members and function to prevent the misfolding of proteins, and the refolding of misfolded proteins, thereby suppressing subsequent protein aggregation and transporting misfolded proteins or aggregates to the ubiquitin–proteasome system for degradation. Considering that Alzheimer’s is a proteinopathy-related disease, studies investigating Hsps in human and mouse brains have revealed altered levels of many family members [32,33,34,35,36]. The ability of PBM to increase levels of Hsps reveals a functionality that may facilitate the refolding of misfolded aggregates of Aβ and tau that contribute to AD pathology and/or the targeted degradation of these aggregates via the ubiquitin–proteosome system. Although the sample size used in this experiment was very small (n = 3), the results are promising and should be further investigated.

Collectively, the studies outlined above demonstrate a role for PBM in modulating levels of Aβ and tau in the brain of animal models of AD. The results of these studies are promising and although they do not outline pathways that facilitate the removal of Aβ and hyperphosphorylated tau from the brain of these animals, there are suggestions that warrant further research.

#### 2.1.3. Effects of PBM on Neurotrophic Factors

In addition to their effects on modulating levels of Aβ and tau in the brain, PBM has also been shown to modulate levels of neurotrophic factors in both cellular and mouse models of AD. Neurotrophic factors are a family of secreted proteins that are required for directing the growth, survival, and differentiation of neurons throughout the central and peripheral nervous systems. Particularly important for brain development and growth are neurotrophins, nerve growth factor (NGF), and brain-derived neurotrophic factor (BDNF). BDNF is crucial for neuronal plasticity and is highly expressed in brain regions such as the hippocampus, hypothalamus, and cortex [37]. Initial studies of BDNF function found it to be a pro-survival protein, supporting neurons during bouts of chronic stress [38]. Subsequent studies have characterized BDNF as harboring anti-apoptotic and antioxidative properties [39,40]. Functions attributed to BDNF are widespread and include facilitating memory formation [41] and enhancing the growth and reorganization of dendritic spines in response to changing neuronal activity [42].

We and others have demonstrated that levels of BDNF mRNA and protein are significantly decreased in the AD brain [43,44,45,46] and have suggested that modulation of endogenous BDNF levels may represent a therapeutic avenue for AD [47,48]. Using low-level laser irradiation (632.8 nm, 10 mW, 12.74 mW/cm^2^) for 0.7, 1.25, 2.5, and 5 min, Meng and colleagues reported increased BDNF expression in cultures of Aβ-treated mouse hippocampal and APP/PS1 hippocampal neurons that was transcriptionally driven via an upregulation of cyclic adenosine monophosphate (cAMP) response element binding protein (CREB) [49]. PBM was also shown to rescue Aβ-induced dendritic atrophy and neuronal death that was characterized by upregulation of a post-synaptic density protein (PSD-95) expression and the increase in length, branching, and spine density of dendrites in hippocampal neurons [49]. Similarly, Heo and coworkers reported increased BDNF expression in the mouse hippocampus following treatment with 660 nm LEDs at a power density of 20 mW/cm^2^ [50]. BDNF upregulation was driven through activation of the extracellular signal-regulated kinase (ERK)/CREB signal transduction pathway [50]. A second study employing the identical wavelength of 632.8 nm reported activation of intracellular inositol 1,4,5-trisphosphate (IP3) receptors, the main Ca^2+^-release channels in the endoplasmic reticulum that resulted in the increase of intracellular Ca^2+^, triggering consequent activation of the ERK/CREB pathway that eventually led to the increase in BDNF expression [51].

Increased expression of other neurotrophic factors, including nerve growth factor (NGF) and glial cell line-derived neurotrophic factor (GDNF), have also been demonstrated following PBM treatment [52,53], although these are less well studied in the realm of Alzheimer’s.

#### 2.1.4. Systemic Effects of PBM

Studies examining the ability of NIR light to penetrate tissues, especially the skull, have raised questions on the feasibility of PBM to affect brain tissue. In one of the early studies addressing this question, Jagdeo and colleagues investigated human cadaver heads and assessed the penetration of 633 nm and 830 nm light, discovering that 11.7% of the 830 nm (3.90 mW/cm^2^) and only 0.7% (0.44 mW/cm^2^) of 633 nm light was able to penetrate both the skin and skull to reach the occipital cortex [54]. Additionally, it was discovered that the amount of penetration also depended on the anatomical region that was being treated, with penetrance of 830 nm irradiation dropping to 2.1% in the frontal lobes and a mere 0.9% over the temporal lobes [54]. Subsequent studies by Tedord and colleagues, also employing human cadavers, compared the penetration capability of 660 nm, 808 nm, and 940 nm light [55]. They found that 808 nm light was best at penetrating both skin and bone of the skull and discovered that this wavelength of light could reach depths in the brain of 4–5 cm [55], important when considering the activation of deeper brain structures such as the hippocampus. In a comparison study that evaluated skull thickness and the ability of NIR light penetration, Lapchak et al. compared the transmission of 780 nm light through the skulls of four different species and found that the mouse skull transmitted ~38% of the light, while for rat skull it was 21%, for rabbit it was 11% and for human skulls it was a mere 4% [56], indicating that ~95% of the signal delivered to the surface of the human skull is attenuated by skin and bone. The results of this study are important as they suggest that results of experiments performed on animals cannot be directly translated to humans due to the large variance in skull thickness, tissue morphology, and fluid characteristics. Attempts at addressing these variations have shown that other routes of delivery may also be feasible.

In pioneering work on this topic, Johnstone and coworkers examined the brains of MPTP (1-methyl-4-phenyl-1,2,3,6-tetrahydro-pyridine)-treated mice, a model of parkinsonism, following 670 nm photobiomodulatory treatment directed at a site distal from the brain [57]. MPTP is a prodrug to the neurotoxin MPP^+^ (1-methyl-4-pyridirium) that selectively targets dopamine (DA)-producing cells in the brain. Following administration of MPTP, which destroys dopaminergic cells in substantia nigra pars compacta (SNc), Johnstone and colleagues irradiated either the head or the body of the mice and analyzed cell survival in SNc. Results revealed that while the effects were not as robust as direct irradiation of the head, treating the body of the animals produced a significant rescue of DA-producing cells in SNc [57]. In a follow-up investigation, this group examined the benefits of pre-conditioning whereby 670 nm NIR light (50 mW/cm^2^, 3 min) was applied to the dorsal surface of animals’ body immediately prior to being administered MPTP and brain tissue analyzed 6 days later [58]. Results revealed that preconditioning animals with PBM resulted in an 85% higher protection of dopaminergic neurons in SNc compared to saline-treated mice [58]. Transcriptomic analysis of midbrain dopaminergic neurons following 10 days of 670 nm NIR treatment (4 J/cm^2^ per day) targeting the dorsum and hindlimbs, followed by MPTP administration and a 7-day survival, revealed differential regulation of a large number of transcripts [59]. Pathway enrichment analysis revealed significant upregulation of genes including stem-cell-related CXCR4 signaling, adipocytokine signaling, oxidative stress response pathways, and those relating to cell proliferation and migration [59]. This study represents one of the first previews of how PBM may exert its neuroprotective action on the brain via remote PBM, and more in-depth studies are warranted.

Focusing on alternate routes of delivery, Fafara et al. targeted mesenchymal stem cells (MSC) by treating bone marrow [28]. They discovered that weekly treatment for 2 months improved cognitive capacity and spatial learning that was accompanied by a 68% reduction in Aβ load in the hippocampus [28]. Pitzschke and colleagues compared the penetration of 671 nm and 810 nm light into cadaver brain that was delivered either via a transcranial or a transsphenoidal approach [13]. Results revealed that the best combination for delivery to the brain was 810 nm NIR light administered via the transsphenoidal route [13]. Intranasal PBM has received wide attention from those concerned with diseases and therapeutics affecting the brain and has been recently reviewed by Salehpour and colleagues [60].

PBM has the potential to treat many pathologies that arise from AD including Aβ load, intracellular tau tangles, neuroinflammation, oxidative stress, decreased trophic factor expression, and metabolic dysfunction. An important advantage of PBM is its noninvasive approach and as such, a patient can be safely treated on multiple occasions if required. PBM can provide sustainable therapeutic benefits including the stimulation of neurotrophic factor release and induce regenerative properties intrinsic to cells. Future research should focus on harnessing the optimal benefits of PBM by establishing parameters required for targeted and even personalized therapy to the brain.

A summary of the results has been provided in Table 1.

## 3. Mildly Invasive

### 3.1. Intestinal Microbe Modulation

The human gastrointestinal (GI) tract consists of a complex neural network that is governed by an arm of the autonomic nervous system referred to as the enteric nervous system (ENS). The ENS is capable of independent regulation of the GI tract, but intestinal microbiota play essential roles in homeostasis, including the conversion of dietary nutrients into active metabolites in the host as well as contributing to neurotransmitter and metabolite levels. Many intestinal disorders such as inflammatory bowel disease (IBD) and irritable bowel syndrome (IBS), as well as psychological and behavioral disorders, have implications in intestinal microbiota dysbiosis [61]. The intestinal microbiota can comprise more than 50 phyla and almost 1000 different species and is known as the enteric nervous system due to its ability to influence the central nervous system via the bidirectional gut–brain–microbiota axis [62]. Intestinal microbial variations have been reported in individuals with Alzheimer’s, with its role in pathogenesis and disease progression yet to be fully delineated [63]. Viral and bacterial infections in general also contribute to Alzheimer’s disease pathogenesis [64], however intestinal microbial alterations do not present with recognizable pathological symptoms in early stages or are overlooked as an indicator of AD-related changes, leading to it being overlooked as a possible therapeutic target. Characterization of human intestinal microbe compositions using 16S rRNA sequencing found marked differences between individuals with mild to moderate AD and individuals with no AD [65]. More than 80 operational taxonomic units (OTUs), or clusters of sequence reads, were found to be disparate from those of non-AD subjects with decreased variety and quantity of OTUs and particular phyla- and genus-level differences seen in AD subjects [65].

#### Intestinal Microbe Involvement in AD

The gut–brain axis is responsible for the intestinal microbiota, possessing the ability to influence neurological functions. Microbiota are responsible for digestive processes via metabolites, including short-chain fatty acids (SCFAs) and gamma-amino butyric acid, that are produced and which then manipulate physiological processes. One example of metabolite regulation by intestinal microbe composition is cholesterol processing and absorption.

Increased plasma cholesterol levels, including high- and low-density lipoproteins as well as total cholesterol, have been proposed as biomarkers or risk factors for AD [66,67,68,69,70]. Usage of statins, drugs utilized to lower serum cholesterol levels, is also linked to decreased AD prevalence [69,71]. Cholesterol arriving in the large intestine may be derived by various mechanisms. Biliary sources contribute an average of between 800 and 1200 mg of cholesterol per day while intestinal mucosal epithelial cell turnover contributes approximately 300 mg per day, and dietary cholesterol not absorbed in the small intestine accounts for nearly 200 mg per day [72,73]. Cholesterol is absorbed in the small intestine across the enterocyte brush-border membrane via protein transporters such as Niemann–Pick C1-like 1 (NPC1L1) and caveolin-1, and proceeds to form intestinal lipoproteins known as chylomicrons in preparation for its movements into blood and lymphatic circulation [74,75]. Serum cholesterol levels are affected by gut microbiota-mediated absorption cholesterol. Approximately half of all cholesterol ingested is absorbed, with the rest being excreted via fecal matter following metabolization by intestinal bacteria [73]. Cholesterol is converted by intestinal microbes to coprostanol, a poorly absorbed saturated molecule which is excreted [76]. This conversion mechanism directly contributes to decreased serum cholesterol levels. Gérard and colleagues have described the genus *Bacteroides* strain D8 as one of the bacterial populations responsible for cholesterol conversion to coprostanol [76]. Studies comparing gut microbial content have identified differences in *Bacteroides* in dementia patients compared to non-AD subjects, although both increases and decreases have been reported in different populations, with decreased levels being more commonly reported [65,77,78,79]. Additionally, metabolites produced by *Bacteroides* have been reported to be pro-inflammatory [80]. *Bacteroides* variations, together with an overall loss in microbial diversity in AD patients [65], would influence the proportion of cholesterol that is absorbed. A study by Zhuang and colleagues that investigated intestinal microbe composition between AD and normal subjects also found a decrease in *Bacteroides* in AD patients, together with other differences in the phylum, class, and family levels [81]. Germ-free mice were also found to have reduced intestinal cholesterol absorption levels coupled with enhanced levels of fecal cholesterol excretion when compared to specific pathogen-free (SPF) mice [82]. Cholesterol malabsorption is directly influenced by gut microbiota composition and integrity and is linked to the neurodegenerative processes of AD [83,84,85].

Mouse models of AD such as APP/PS1 mice also have differing intestinal microbe compositions compared to wild-type mice, with differences seen even prior to the appearance of Aβ plaques [86]. Interestingly, APP/PS1 mice were found to have an elevated alpha diversity (within-samples diversity) as well as a more diverse and varied microbiota composition when compared to wild-type mice at 9 months of age, although with significant differences in composition commencing from 2 months [86]. This study also found increased reactive gliosis with more ramified and clustered microglia surrounding Aβ plaques in APP/PS1 mice, highlighting the link between microbial dysbiosis and neuroinflammation [86]. Increased alpha diversity was also reported in an earlier study involving APP/PS1 mice by Harach et al. and was further correlated with increased cortical Aβ levels [87]. Harach and colleagues studied germ-free APP/PS1 mice that completely lacked intestinal microbiota and found significantly reduced cortical Aβ levels compared to conventionally bred mice [87]. Long-term antibiotic treatment of APP/PS1 mice also decreased Aβ deposition [88]. Although there was increased antibiotic resistance among the intestinal microbes of mice exposed to long-term antibiotic treatment, levels of soluble Aβ (the more degradable form) were increased in the treated animals [88], probably indicative that the resistant microbes were essential for plaque solubilization. Furthermore, microglial reactivity surrounding amyloid plaques was found to be decreased, although the quantity of microglia relative to amyloid plaque size remained unchanged [88]. Interestingly, antibiotic treatment in wild-type mice was found to cause generalized memory loss and decreased hippocampal neurogenesis which could not be rescued with reconstitution of intestinal microbiota but was restored with probiotic administration or exercise [89]. These effects were mediated by Ly-6C [89], a monocyte/macrophage and endothelial cell differentiation antigen that is regulated by interferon gamma.

It has been hypothesized that intestinal microbial dysbiosis commences prior to mild cognitive impairment and remains unchanged through disease progression to AD [79]. This was based on the comparison of MCI and AD patient blood and fecal microbe composition against controls as well as the association of cognition with microbial composition. The relative abundance of genera *Bacteroides* was decreased, and *Escherichia* and *Lactobacillus* were increased compared to controls but remained similar between MCI and AD subjects [79]. This led the authors to suggest that intestinal dysbiosis may function as a biomarker of AD for patients presenting with MCI together with other biomarkers such as Aβ levels [79]. A preclinical study employing the APP/PS1 mouse model of AD compared the longitudinal makeup of intestinal microbiota between transgenic and wild-type mice and reported that differences in composition commenced at 6 months of age [90]. In particular, the genus *Sutterella* of the Proteobacteria group, which is increased in autism disorder and the inflammatory *Erysipelotrichaceae* family, were increased [90].

Inflammatory mechanisms play an important role in AD with reactive gliosis and astrocytosis commonly reported in AD brains with increased pro-inflammatory cytokines such as interferons (IFN) and interleukins (IL) [7,91,92,93,94]. Aβ, despite being a pathological hallmark of AD, is also an antimicrobial peptide and hence, increased Aβ has been proposed to be an immune response to infectious agents [95]. For example, knockout of the type-1 IFN α-receptor in APP/PS1 mice reduced Aβ monomers, attenuated microgliosis, and increased the astrocytic response while amyloid deposition remained unaltered [91]. Interleukins are capable of influencing inflammatory mechanisms; for example, the pro-inflammatory IL-1β increases non-amyloidogenic cleavage of APP while decreasing amyloidogenic cleavage and hence reducing Aβ plaque deposition [96,97]. Expression of IL-4, an inducer of the M2a microglial phenotype associated with repair and protection, in the frontal cortex of APP/PS1 mice via an AAV vector was shown to produce a trend toward a decrease in amyloid deposition, although the study had to be terminated early due to increased mortality in the mice [98]. In a comparable study by Kiyota and colleagues, sustained IL-4 expression via an AAV vector injected into the hippocampus of APP/PS1 mice resulted in reduced astro- and microgliosis, Aβ oligomerization and deposition, and improved spatial memory [99]. Contradicting these results, Chakrabarty and colleagues reported increased plaque deposition and decreased soluble Aβ_40_ internalization by microglia in TgCRND8 mice [100]. The main difference between these three studies was the age at which the IL-4-expressing AAV vector was introduced into the system and this may highlight an important variable when considering therapeutic strategies. Latta and colleagues administered the vector when the APP/PS1 mice were 3 months of age while in the Minter and Chakrabarty studies, APP/PS1 and TgCRND8 mice received the IL-4-expressing vectors at 9 months of age, when plaque deposition was well established. The results of these three studies reveal that the administration of IL-4 late in disease is not beneficial whereas delivery early in disease may in fact have therapeutic effects on amyloid plaque deposition and cognition.

To examine the capability of intestinal infections to alter immune responses elsewhere in the body, which ultimately affect the brain, Montacute and colleagues infected 3xTg-AD mice, a model of Alzheimer’s disease, with different parasites and monitored the subsequent inflammatory response in the intestines and brain [101]. Cortical microglial activation as well as splenocyte and mesenteric lymph node cytokine and chemokine levels were increased in infected 3xTg-AD mice compared to controls [101]. Similarly, intestinal microbial compositions were shown to be altered in patients with amyloid loading who also displayed increased pro-inflammatory and decreased anti-inflammatory cytokines that corresponded in particular to an increase in pro-inflammatory bacteria from the *Escherichia/Shigella* genus [102]. Patients with amyloid loading also had decreased *Eubacterium rectale*, which exerts anti-inflammatory effects [102].

Hence, multiple lines of evidence suggest that intestinal microbial dysbiosis is a viable therapeutic target. Further, studies have commenced to prove the efficacy of reducing dysbiosis and/or restoring intestinal microbial composition against AD.

### 3.2. Restoration of Intestinal Microbiota to Reduce AD Symptoms

#### 3.2.1. Anti- and Probiotics

Broad-spectrum antibiotics are designed to elicit non-specific, wide-spread destruction of bacterial populations, in particular working to eliminate the growth of groups of bacteria. Hence long-term antibiotic administration may cause adverse effects to intestinal microbes, reducing their diversity and stability. Ampicillin administration was found to cause colon inflammation, impair memory functions, and cause anxiety-related behavior in rats, all of which were rescued by the probiotic *Lactobacillus fermentum* [103]. However, antibiotic administration has had varied results in clinical trials, with some studies showing significant improvements in AD patients and some showing no change (reviewed by [104]). A synbiotic comprising three probiotics, *Lactobacillus plantarum*, *Lactobacillus fermentum*, and *Bifidobacteria longum*, administered to a Drosophila melanogaster model of AD resulted in higher survivability, increased motility, decreased Aβ, and restored acetylcholinesterase activity following 30 days of treatment [105].

In rodent models of AD, Lee and colleagues orally administered the probiotic *Bifidobacterium longum* to 5xFAD mice to investigate its effects in vivo following observations of its efficacy in reducing inflammation in the BV-2 cell line [106]. *Bifidobacterium longum* was shown to alter intestinal microbial composition while impeding bacterial lipopolysaccharide production [106]. Cognitive function was also rescued while inflammatory cytokines TNF-α and IL-6 and microglial activation in the hippocampus were inhibited in the treated mice [56,106]. The probiotic *Bifidobacterium breve* strain A1 was also tested orally in AD mice and was found to restore cognitive function, increase intestinal carbohydrate metabolism, and regulate gene expression to reduce excessive immune responses to Aβ, all of which contributed to reduced AD pathology [107].

A randomized, double-blind, placebo-controlled trial by Akbari and colleagues investigated the effects of treating AD patients with probiotic milk containing *Lactobacillus acidophilus*, *Lactobacillus casei*, *Bifidobacterium bifidum*, and *Lactobacillus fermentum* daily for 12 weeks. Treated AD patients showed significant improvement in the Mini-mental State Examination and significant decreases in the oxidative stress marker malondialdehyde (MDA) and serum high-sensitivity C-reactive protein (hs-CRP), an inflammatory marker, with no change in total cholesterol but improved insulin functioning (QUICKI) [108]. A second randomized double-blind trial by the same group investigated the effects of a different probiotic cocktail containing selenium, an essential trace mineral, with *Lactobacillus acidophilus*, *Bifidobacterium bifidum*, and *Bifidobacterium longum* against selenium only and also controls receiving placebo [109]. Supplementation of probiotics with selenium resulted in improved MMSE scores, reduced hs-CRP, and reduced insulin and triglyceride levels, among other markers of disease, as well as some metabolic profiles. [109].

A study employing a cocktail of probiotics consisting of *Lactobacillus case*, *Lactococcus lactis*, *Lactobacillus acidophilus*, *Bifidobacterium lactis*, *Lactobacillus paracasei*, *Lactobacillus plantarum*, *Bifidobacterium lacti*, *Bifidobacterium bifidum*, and *Lactobacillus salivarius* administered to AD patients for 28 days revealed numerous beneficial effects of the supplement [110]. Levels of zonulin, a regulator of intercellular tight junctions and a biomarker of intestinal barrier function [111], was significantly decreased while anti-inflammatory *Faecalibacterium prausnitzii* levels were increased following treatment [110]. Additionally, levels of serum kynurenine, a metabolite of the amino acid l-tryptophan and one that orchestrates local and systemic responses to control inflammation [112], was also increased [110], indicative of the anti-inflammatory properties of the probiotics. In preclinical studies, Wistar rats receiving intracerebroventricular Aβ injections were administered the probiotics *Lactobacillus acidophilus*, *Bifidobacterium bifidum* and *Bifidobacterium longum* via intragastric gavage which were once again found to restore cognitive function and increase hippocampal long-term potentiation, hence enhancing synaptic plasticity and transmission [113]. Aβ plaques also appeared to be eliminated following probiotic treatment with decreased plasma malondealdehyde levels and increased colony forming units of fecal material, showing increased integrity of fecal microbes [113]. This as well as other studies have raised the possibility that a regular dose of probiotic may in fact be beneficial for human health and warrants further investigation.

#### 3.2.2. Fecal Microbe Transplants

Fecal microbiota transplantation (FMT) is a therapeutic method in which fecal matter from healthy donors is transplanted to individuals with disease in an effort to stabilize or normalize intestinal microbial populations and function in the recipient. The method was pioneered for use in diseases of the gut and is employed successfully for the treatment and management of *Clostridium difficile* infection, colitis, irritable bowel syndrome, as well as neurologic and autoimmune disorders [114,115,116,117,118]. Importantly, FMT has been demonstrated to be a safe and feasible long-term treatment in humans [114,115].

Recently, FMT has shown efficacy in reducing AD pathology in AD mice models [119,120,121]. Fecal transplants from wild-type donor mice to those in a robust 5xFAD mouse model study of AD were shown to significantly improve cognitive performance and decrease plaque size and number in treated mice [119]. Interestingly, the age of the donor mice played a significant role in the effects observed. FMT from 10–12-week-old donor wild-type mice resulted in greater improvements in cognition and lower amyloid load in recipient 5xFAD mice compared to transplants received from 30–32-week-old wild-type littermates [119]. FMT employed in another AD mouse, the APP/PS1 mouse, also revealed similar findings where improvements in cognition and a decrease in Aβ deposition were reported [68,120]. Additionally, increases in synaptic plasticity and decreases in the neuroinflammatory mediators COX-2 and CD11b were observed in APP/PS1 mice that received FMT compared to controls. Examining the fecal microbe composition of the treated mice revealed alterations in the treated APP/PS1 mice [120], demonstrating that FMT does in fact alter the gut microbiome of the recipient. Importantly, SCFA butyrate levels were found to be significantly replenished in treated mice, once again demonstrating the efficacy of FMT in altering gut microbial composition and exerting neuroprotective effects [120].

Studies investigating the composition of the gut microbiome in wild-type mice identified an age-based association in healthy C57BL/6 mice [121]. This study by D’Amato and colleagues found that younger mice transplanted with fecal microbiome from older mice acquired a microbial population profile resembling that of the aged donors [121]. Complementing this profile, the younger mice showed impaired spatial learning, although anxiety levels and motor learning were unaffected [121]. Further, expression of proteins associated with learning and neurotransmission were downregulated in the hippocampus of young mice receiving a fecal transplant from older donors [121]. Intriguingly, intestinal permeability, cytokine expression, astrocytosis, and overall neuroinflammation were found to be unchanged, suggesting that inflammatory pathology is unique to AD [121]. A case report of recovery of an Alzheimer’s patient who received FMT for a *Clostridioides difficile* infection is the only human study reported to date [122]. Here, an 82-year-old male with an MMSE score of 20 received FMT from his 85-year-old, cognitively astute wife and at his 2-month follow-up visit showed improvements in cognition and scored 26 on the MMSE which improved to 29 at his 4-month visit [122].

A recent growing interest in the role of the gut microbiota in the pathogenesis of AD has resulted in mixed levels of excitement with gut microbiota also known to exude amyloid and lipopolysaccharides that could activate pathogenic signaling pathways involved in neurodegeneration or neuroinflammation via the gut–brain axis [123,124]. Intestinal microbial composition and integrity has surfaced as a critical tool with potential as a therapeutic target against AD [125,126] and other neurodegenerative disorders. Improving or altering intestinal microbial composition employing probiotics, antibiotics, or fecal microbiota transplants has been shown to elicit neuroprotective effects and reduce AD pathology. Furthermore, intestinal microbial dysbiosis could be detected in advance via fecal screening mechanisms, prior to the development of AD symptoms, and therefore could serve a dual role as both a biomarker and a therapeutic target. Increased investigation into exact mechanisms of interaction between intestinal microbiota and the central nervous system would aid to advance the understanding of intestinal microbial dysbiosis as a causative factor of AD and increase its potential as a therapeutic target.

A summary of the results has been provided in Table 1.

## 4. Invasive

### 4.1. Deep Brain Stimulation

DBS involves the use of surgically implanted electrodes in the brain to deliver electrical pulses [127]. The exact mechanisms underlying DBS are still being elucidated but many theories suggest these electrical pulses have a neuromodulatory effect by either disrupting pathological brain circuit activity or by inducing regenerative effects in the brain [128,129]. Many in vitro studies have demonstrated therapeutic effects of electrical stimulation on cells of the nervous system such as electrotaxis, proliferation, differentiation, cell enlargement, neurite extension, and increased expression of neurotrophic factors [130,131,132,133].

The idea of neurostimulation or neuromodulation originated in the 1930s when surgeons investigating optimal targets for ablation began stimulating deep brain regions [134]. Subsequent research investigating the treatment of motor disorders discovered that while low-frequency stimulation appeared to worsen symptoms, higher frequencies (130–200 Hz) were capable of suppressing motor disturbances [135]. One of these brain regions was the subthalamic nucleus, a current key target in the treatment of Parkinson’s disease (PD) [134]. Research and use of DBS waned following the introduction of levodopa for the treatment of movement disorders associated with PD but a few continued DBS research for patients who did not benefit from levodopa treatment [134]. The DBS research field grew alongside the technological development of the neurostimulator [134]. Prior to the design of neurostimulators, modified pacemakers were used for neurostimulation [134] that included the placing of stimulators near the spinal cord to treat chronic pain [136]. The positive results from modified pacemakers drove innovation of neurostimulators designed as they are today. DBS was first approved by the Food and Drugs Administration for treating PD and is currently widely used for PD [134] when other avenues of treatment have been exhausted.

### 4.2. Site of Stimulation

Following positive outcomes in PD, DBS was trialed for other disorders such as obsessive-compulsive disorder [137] and obesity [138]. A case study involving the use of bilateral hypothalamic DBS for obesity yielded unexpected results when the patient exhibited improved visuospatial and verbal memories and could recall detailed autobiographical memories [138]. Further investigation led to the identification of the fornix as a potential site for DBS to treat AD and subsequently Phase I and II clinical trials have shown promising results [139,140].

Since then, other potential locations for electrode placement for AD have been studied and include the entorhinal cortex [141], nucleus basalis of Meynert [142], vertical limb of the diagonal band [143], and the central capsule/ventral striatum [144]. Each of these sites can be linked to a memory circuit that is affected by AD pathology [129,145]. The mechanisms for each stimulation site may be different but understanding the mechanisms for each site may be invaluable, especially to target specific symptoms [129].

### 4.3. Mechanisms

The literature is replete with both animal studies and human trials that have reported improvements in cognitive function following treatment with DBS. The major theories underpinning these improvements include the reduction in amyloid load in the AD brain, neurogenesis, increased cerebral glucose metabolism, neurotrophic factor expression, and modulation of synapses to facilitate neurotransmitter release [146,147].

#### 4.3.1. Reduction in AD Pathology

Studies employing DBS have been proven to counteract the accumulation of Aβ, neuroinflammation, and neurodegeneration. Mann and colleagues used chronic high-frequency (130 Hz) stimulation focused at the entorhinal cortex of 3xTg mice and reported significantly improved cognitive functions [148]. The authors attributed the improvement to decreases in Aβ plaques, Aβ42 levels in hippocampal subfield CA1, and the wider hippocampus as well as cortical levels of total tau. There was also evidence of neurogenesis in the dentate gyrus.

Another study used high-frequency stimulation of the thalamus in TgCRND8 mice, another model of AD that expresses fivefold higher APP levels resulting in early plaque formation combined with cognitive deficits, and reported improved short-term memory [149]. Results from ex vivo stimulation of hippocampal slices showed improved short-term potentiation in wild-type mice receiving 50 and 100 Hz stimuli, whereas a higher frequency of 200 Hz was required to produce similar effects in hippocampal slices of TgCRND8 mice [149]. As well as changes in synaptic activity, the authors also observed changes in α- and β-secretase activity that appeared to be modulated by frequency. While low-frequency signals (0.2 Hz) decreased activities for both secretases compared to non-stimulated slices used as baseline controls, a stimulus of 50 Hz increased α-secretase activity nearly twofold in the CA1 hippocampal subfield in TgCRND8 mice [149]. Smaller, but significant changes were also observed in α-secretase activity at the higher stimulus intensities of 100 and 200 Hz. Although there were marginal changes in β-secretase, the results demonstrated that high-frequency stimulation selectively increased α-secretase activity levels in these mice. The cleaving of APP via α-secretase leads to a non-amyloidogenic generation of proteolytic products of APP compared to β-secretase cleavage, which generates the precursor of Aβ [150]. Although β-secretase does lead to the generation of Aβ, the presence of physiological quantities of the enzyme is important for proper synaptic, emotional, and cognitive function [151]. While levels of Aβ were not measured, it is possible that changes in secretase activity would alter the generation and subsequent accumulation of Aβ in plaques.

In a study employing a rat model of AD, Leplus et al. treated 18-month-old TgF344-AD rats and aged match controls with high-frequency DBS treatment for 5 weeks [152]. Immunostaining revealed both Aβ/APP and Aβ42 levels were significantly reduced in both the hippocampus and cortex of stimulated AD rats compared to non-stimulated controls. Neuroinflammation is commonly seen in AD brains and DBS may be able to target this pathophysiology. Iba-1 levels, a marker of activated microglia, were significantly decreased in the hippocampus and cortex of treated AD rats compared to non-treated AD controls [152]. Furthermore, treated AD rats had comparable Iba-1 levels to wild-type rats. Similar results were also observed with GFAP staining (astrocytes), with statistically significant decreases observed in the treated AD rats compared to untreated controls. The study also compared DBS in wild-type rats and reported no difference in Iba-1 or GFAP immunoreactivity between treated and non-treated WT rats, suggesting that DBS does not contribute to neuroinflammation [152].

#### 4.3.2. Effect of DBS on Brain Neurotrophic Factor Release

Patients with AD are known to exhibit significantly decreased levels of trophic factors such as nerve growth factor (NGF) and brain-derived neurotrophic factor (BDNF) [43,44] that are believed to contribute to the degeneration of neurons. Using the intraventricular 192 IgG-saporin model of AD in rats, Jeong and colleagues treated these animals with DBS of the medial septum, either 5 days before the acquisition phase of the Morris water maze (pre-stimulation group), for 5 days during the acquisition (training stimulation group), or 2 h before the probe test [153]. The largest improvements were reported in the pre-stimulation group for scores of spatial tests, where not only was there a significant improvement compared to non-stimulated rats but the pre-stimulation group performed similarly to rats without lesions [153]. Significant improvements were also observed in the training stimulation group. Proposed mechanisms of action included changes in BDNF and neurogenesis. Significantly increased expression of BDNF was observed in the frontal cortex of all stimulation groups as well as in the hippocampus, although increases here were not statistically significant [153]. Probe stimulation resulted in the highest increase in BDNF compared to the other stimulation paradigms, but the authors suggested that the most improved measure of memory was seen in the pre-stimulation group as increases here were sustained for the longest duration. Additionally, the pre-stimulation group showed the highest expression of Sox2 and doublecortin (DCX), suggesting that this group experienced the highest levels of neurogenesis.

Examining deep brain structures that may alleviate memory deficits in AD patients, Gondard and coworkers stimulated the fornix of rats for one hour and analyzed protein expression 1 h, 2.5 h, 5 h, and 25 h following stimulation [154]. The fornix is a white matter tract that connects the hippocampus to structures such as the thalamus, hypothalamus, and basal forebrain among others. It is considered part of the limbic system and has been associated with learning and memory. DBS of the fornix for 1 h resulted in a significant 2.3-fold increase in BDNF expression in the hippocampus [154]. Significant changes in VEGF were also observed in the hippocampus at 2.5 h following stimulation. Additional changes observed at the 2.5 h time point included significant increases in the immediate early gene cFOS, GAP-43, synaptophysin, and α-synuclein. GAP-43 and synaptophysin are synaptic proteins important in the proper establishment of neural circuitries and are implicated in regenerative activities such as synaptogenesis and axonal growth [155,156]. At the 5 h time point, levels of most of these proteins returned to baseline, suggesting that although the activation of these proteins was rapid, their increases were transient. Additionally, there were no changes of GDNF and the AD-related proteins tau, ptau, APP, or chaperone proteins such as HSP70 and HSP40 [154].

#### 4.3.3. Effect of DBS on Levels of Cerebral Metabolism

Numerous lines of evidence suggest that neurodegenerative diseases are linked to or caused by metabolic deficiencies [157,158,159]. For example, hypometabolism of glucose in the cerebrum is often exhibited in patients presenting with AD [140]. A randomized, double-blind stage II clinical trial involving bilateral DBS of the fornix in 42 mild AD patients led to increased cerebral glucose metabolism [139]. Patients receiving stimulation showed increased metabolism at 6 months, but this was not significant at 12 months. Interestingly, the greatest effect was observed in patients over the age of 65. The study supports the theory that DBS works by disrupting pathological brain circuit activity and improving neural activity by activating axons of the fornix [139]. A case study in 1985 reported that repetitive cycles of DBS employing an electrode implanted in the left basal nucleus of Meynert of a 74-year-old patient with senile dementia of Alzheimer’s type (SDAT) for 9 months resulted in preserved cortical glucose metabolic activity in the ipsilateral temporal and parietal lobes while activity declined in other locations of the cortex [160]. Although this increase in metabolic activity failed to eventuate in clinical improvement, the results demonstrated the efficacy of DBS in maintaining and/or restoring metabolic activity in the brain. In an unrelated study employing DBS of the fornix for one year, the investigators reported increased glucose metabolism as well as improved cognitive function [161].

In a phase I trial in six patients with mild AD, continuous forniceal DBS for 12 months was shown to drive neural activity in areas including memory circuits such as the entorhinal and hippocampus as well as the parietal, primary sensory, and motor regions and cerebellum [140]. PET scans showed an early reversal (within 1 month) of the impaired glucose utilization in the temporal and parietal lobes that was maintained after 12 months of continuous stimulation. Evaluation of cognition also revealed improvements and a slowing in the rate of cognitive decline at 6 and 12 months [140].

In another study investigating the effects of forniceal DBS in six individuals with AD, Sanker and colleagues found changes in hippocampal volume and levels of degeneration [162]. In two out of six treated patients, bilateral increases in hippocampal volume were observed with volume increases persisting 3 years following treatment in one of these individuals [162]. DBS was also found to slow hippocampal atrophy compared to 25 age-, sex-, and severity-matched AD controls. Increases in hippocampal volume were associated with changes in hippocampal metabolism and with volume changes in both mammillary bodies and fornix. These results suggest that DBS has a circuit-wide effect. Additionally, there are numerous studies that have provided evidence supporting the presence of neurogenesis following DBS and have purported that neurogenesis may underpin improved cognitive function [148,152,153].

#### 4.3.4. Timing of Intervention

There is mounting evidence that DBS has benefits for treating AD. However, it is important to establish a window during the progression of the disease that is most effective for intervention. For example, is it possible for DBS to improve a progressed AD patient or would it more beneficial as a preventative measure? Xia and colleagues have shown that a high-frequency (130 Hz) DBS signal delivered for one hour to the entorhinal cortex of TgCRND8 mice at 6 weeks (young) and 6 months (old) has the potential to mitigate disease progression and could, to some extent, be used to prevent disease progression [141]. Memory impairments in these mice develop at 6 weeks and precede plaque deposition, which commences at 10–12 weeks. Memory correlates were measured 1, 3, or 6 weeks following treatment. Analysis of contextual fear conditioning showed substantial improvements in young mice trained 3 weeks following stimulation. Results were indiscernible from those of WT mice when training was performed 6 weeks following DBS treatment, demonstrating long-lasting effects of the stimulation. Entorhinal cortex DBS also reversed spatial learning deficits in both young and old TgCRND8 mice, once again demonstrating that the method can have effects even during later stages in the disease. The changes in the old mice were not related to plaque load as these were essentially unchanged [141]. This would suggest that although DBS is not able to reverse plaque load in the brain, it can still exert its effects on memory correlates and demonstrates the potential of DBS as an effective treatment for both early and later stages of disease progression.

An intriguing finding from the animal studies described above is the observation that cognitive improvements following DBS often appear after a delayed period of time. For example, Xia and colleagues found no cognitive improvements when training and testing were performed 1 week following DBS but reported substantial changes at 3 weeks and significant changes at 6 weeks. Similarly, Jeong and coworkers found that the greatest improvements in rat spatial memory were observed in a group that received stimulation 5 days prior to the acquisition phase of the memory task, and hence 11 days pre-test compared to 5 days or 2 h pre-test [153]. Expression of BDNF was significantly increased in the frontal cortex and increases were also reported in the hippocampus of rats receiving DBS [153], probably suggesting that the increase in BDNF underpinned structural changes to pathways associated with memory and cognition that required time for remodeling. It may be argued that the results of the investigation by Godard and colleagues, who reported maximal expression of trophic and synaptic proteins 2.5 h following treatment [154], contradict the remodeling hypothesis proposed above. However, it must be noted that the Godard study only examined protein expression up to 5 h following stimulation and as such, longer periods of protein expression followed by studies examining structural changes are warranted. It is also plausible that chronic treatment can lead to a cumulative regenerative effect of the upregulated proteins.

Although an invasive technique, DBS has the advantage of providing long-lasting and sustainable effects. Similar to PBM, DBS could provide a sustainable method of introducing BDNF and other trophic supports in specific regions in the brain that can treat various symptoms of AD. Evidence suggests that DBS can slow disease progression and even exert an impact late in the course of disease.

A summary of the results has been provided in Table 1.

**Table 1:** A summary of the three different non-pharmacological therapeutic intervention avenues that have been studied for the treatment of Alzheimer’s disease.

**Noninvasive**			
**Photobiomodulation**	CCO	Rat postnatal visual cortex neurons	670 nm and 880 nm [20]
		Human adipose-derived stem cells	660 and 810 nm [21]
		Genetically modified Cox10 cell lines	660 nm [24]
	Aβ and tau	12-month-old hTau mice and 3xTgAD mice	670 nm, 4 J/cm^2^ of NIR light for 90 s daily, 5 days/week for 4 weeks [12]
		2- and 6-month-old 5xFAD mice	610 nm, 1.7 mW/cm^2^; 2.0 J/cm^2^ for 20 min daily, 3 times/week for 14 weeks [29]
		APP transgenic mice	2830 mW/cm^2^, 566 mW/cm^2^ 5660 mW/cm^2^ [14]
		5-month-old APP/PS1 mice and 7-month-old K3 mice	670 nm, 44 mW/cm^2^ of NIR light for 90 s daily, 5 days/week for 4 weeks [30]
		TASTPM mice	1072 nm at 600 Hz, 5 mW/cm^2^ for 6 min daily, 2 days/bi-weekly for 5 months [31]
	Neurotrophins, NGF, BDNF, IP3	Aβ-treated and APP/PS1 mouse hippocampal neurons	632.8 nm, 10 mW, 12.74 mW/cm^2^ for 0.7, 1.25, 2.5, and 5 min [49]
		Hippocampal Ht-22 cell line	660-nm 20 mW/cm^2^ [50]
		Cultured dorsal root ganglion neurons (DRGNs)	632.8 nm [51]
	Distal site	MPTP-induced PD mice	670 nm via body [57]
		Human cadaver brain	671 nm and 810 nm via transcranial or transsphenoidal [13]
**Mildly Invasive**			
**Anti- and probiotics**	Lactobacillus and bifidobacterium genus	Ampicillin-treated rats	Probiotic *Lactobacillus fermentum* Colon inflammation rescue [103]
		Drosophila melanogaster AD model	Synbiotic of 3 probiotics *Lactobacillus plantarum*, *Lactobacillus fermentum* and *Bifidobacteria longum* Increased survivability and motility, decreased Aβ [105]
		5xFAD mice	Probiotic *Bifidobacterium longum* Cognitive function rescue and inflammatory cytokines inhibition [106]
		ddY mice AD model	Probiotic *Bifidobacterium breve* Restored cognitive function, regulation of metabolism, gene expression, and immune responses [107]
		AD patients	Probiotic milk with *Lactobacillus acidophilus*, *Lactobacillus* casei, *Bifidobacterium bifidum*, and *Lactobacillus fermentum* Cognitive improvement and decreased inflammatory markers [108]
		AD patients	Probiotic cocktail with selenium, *Lactobacillus acidophilus*, *Bifidobacterium bifidum*, and *Bifidobacterium longum* Improved cognition and reduced insulin and triglyceride [109]
		AD patients	Probiotic cocktail with *Lactobacillus case*, *Lactococcus lactis*, *Lactobacillus acidophilus*, *Bifidobacterium lactis*, *Lactobacillus paracasei*, *Lactobacillus plantarum*, *Bifidobacterium lacti*, *Bifidobacterium bifidum*, and *Lactobacillus salivarius* Increased anti-inflammatory response [110]
		Wistar rats AD model	Probiotics *Lactobacillus acidophilus*, *Bifidobacterium bifidum*, and *Bifidobacterium longum* Restored cognition [113]
**Fecal microbiota transplants**	Mice	5xFAD mice	Improved cognition and decreased Aβ [119]
		APP/PS1	Improved cognition and decreased Aβ [120]
	Human	AD patient	Improved cognition [122]
**Invasive**			
**Deep brain stimulation**	High frequency	3xTg mice	130 Hz at entorhinal cortex, improved cognition [148]
		TgCRND8 mice	50 and 100 Hz at thalamus, improved short-term memory [149]
		TgF344t rats	130 Hz at fornix, decreased Aβ [152]
		Wister rats	130 Hz at fornix, increased hippocampal BDNF expression [154]
		42 mild AD patients	130 Hz at fornix, increased cerebral glucose metabolism [139]
		6 mild AD patients	130 Hz at fornix, increased neural activity, [130] and increased hippocampal volume [162]
	Mid frequency	Sprague-Dawley rats	60 Hz at medial septum, improved spatial memory [153]
	Mid and low frequency	TgCRND8	0.2 Hz at hippocampus, decreased secretase activity 50 Hz at hippocampus, increased α-secretase activity [149]

## 5. Conclusions

AD represents an impending health crisis requiring urgent attention. The above-discussed methods are a non-exhaustive list of therapeutic strategies that have shown potential in mitigating or possible reversing AD. AD has been proven to involve numerous pathogenic mechanisms across multiple systems, making it a challenge to discover effective therapeutic targets. In order to develop an effective treatment against AD, the integration of different approaches targeting the various pathways is required. Hence, more investigation of cross-system interactions and their effects on AD pathology is needed.
